# Linezolid-resistant and vancomycin-resistant *Enterococcus faecium* urinary isolate in a pediatric B-ALL patient

**DOI:** 10.1128/asmcr.00085-25

**Published:** 2025-10-22

**Authors:** Emma Seevak, Tanvi S. Sharma, Alaric W. D'Souza

**Affiliations:** 1 Harvard Medical School 1811 , Boston, Massachusetts, USA; 2 Department of Infectious Diseases, Boston Children’s Hospital 24172 , Boston, Massachusetts, USA; 3 Department of Immunology and Infectious Diseases, Harvard T.H. Chan School of Public Health 1857 , Boston, Massachusetts, USA; Rush University Medical Center, Chicago, Illinois, USA

**Keywords:** immunocompromised, pediatrics, urinary tract infection, vancomycin resistance, antibiotic resistance, linezolid, VRE

## Abstract

**Background:**

Vancomycin-resistant *Enterococcus faecium* (VRE) is a major cause of healthcare-associated infections, especially in immunocompromised hosts. Linezolid is a key therapeutic agent due to its oral bioavailability and activity against resistant Gram-positive bacteria. While rare in U.S. pediatric patients, linezolid resistance can severely limit treatment options.

**Case Summary:**

We describe a 16-year-old female with high-risk B-cell acute lymphoblastic leukemia whose hospitalization was complicated by urinary tract infection with VRE. Serial isolates tested on multiple antimicrobial susceptibility testing platforms yielded discordant results for linezolid susceptibility. Minimum inhibitory concentrations to linezolid and chloramphenicol increased together, suggesting potential ribosomal-target-mediated resistance. She ultimately required daptomycin therapy for linezolid-resistant VRE urinary tract infection treatment.

**Conclusion:**

This case underscores the diagnostic challenges in detecting emerging linezolid resistance in *E. faecium*, particularly in immunocompromised patients. Accurate, timely susceptibility testing and improved access to confirmatory or molecular diagnostics are essential to guide therapy for VRE where linezolid remains one of the few viable therapeutic options.

## INTRODUCTION

*Enterococcus faecium* is a leading cause of nosocomial infections, particularly among immunocompromised patients, and has developed resistance to many standard therapies (1, 2). Vancomycin-resistant *E. faecium* (VRE) presents a significant management challenge, and linezolid is frequently used for treatment (3–6). Linezolid is an oxazolidinone antibiotic that inhibits bacterial protein synthesis by binding to the 50S ribosomal subunit at the peptidyl transferase center, preventing formation of the initiation complex essential for translation (7–9). Linezolid and daptomycin are common alternatives to vancomycin for resistant Gram-positive infections, but linezolid’s oral bioavailability and central nervous system and lung penetration are strengths over daptomycin (10–12).

Multiple mechanisms can confer linezolid resistance in Gram-positive organisms, including (i) ribosomal mutations in 23S rRNA and/or L3/L4/L22 ribosomal proteins, (ii) loss of the *rlmN* gene (which encodes a RNA methyltransferase), (iii) efflux pumps, (iv) Cfr and Cfr-like methyltransferases, and (v) ribosomal protection proteins (OptrA, PotxA, and PotxA2) (13). Though still relatively uncommon, linezolid resistance is increasing (14), particularly in oncology and transplant populations with high antimicrobial exposure and healthcare contact (15). Outbreaks of linezolid-resistant VRE have been reported in U.S. academic medical centers, including in patients without prior linezolid exposure (16, 17). Pediatric reports of linezolid resistance remain rare, but early cases highlight the risk of losing one of the few reliable oral antimicrobials for serious Gram-positive infections (18).

Detection of emerging resistance is complicated by variability between testing platforms, such as automated systems (e.g. VITEK 2 [bioMérieux], BD Phoenix [BD]) and manual methods (e.g. ETEST) (19). Both VITEK 2 and Phoenix use broth microdilution-based methodologies to infer minimum inhibitory concentrations (MICs) from growth kinetics, but differences in panel design, inoculum preparation, and algorithmic interpretation can produce discrepancies—particularly near clinical breakpoints (20, 21). These challenges complicate clinical decision-making and underscore the importance of confirmatory testing in cases of suspected resistance.

The urinary tract represents a potential site for rapid selection of resistant organisms, especially under prolonged antibiotic pressure and in immunocompromised hosts (22, 23). We present a case of linezolid- and vancomycin-resistant *E. faecium* urinary tract infection in an immunocompromised adolescent, illustrating the challenges of detecting evolving resistance, reconciling discordant susceptibility results, and selecting effective therapy.

## CASE PRESENTATION

A 16-year-old female with spina bifida, mild developmental delay, and polycystic ovarian syndrome was diagnosed with high-risk B-cell acute lymphoblastic leukemia (B-ALL) 3 months prior to hospitalization. She initiated therapy per Children’s Oncology Group AALL1732 protocol, Arm A, consisting of induction (cytarabine intrathecal [IT], vincristine intravenous [IV], daunorubicin IV, pegaspargase IV, methotrexate IT, corticosteroids) and consolidation (cyclophosphamide IV, cytarabine IV, mercaptopurine orally, methotrexate IT, vincristine IV, and asparaginase IV).

**Fig 1:**
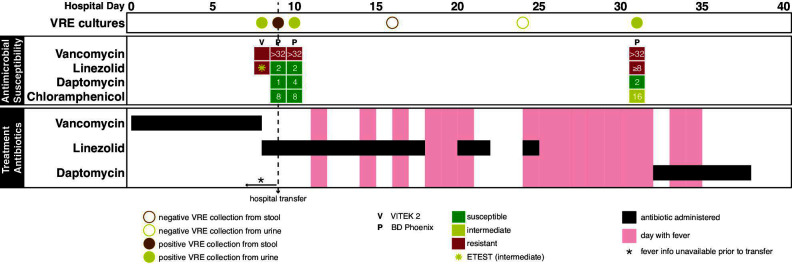
*Enterococcus faecium* cultures, antimicrobial susceptibility testing, and resistant Gram-positive antibiotic use during the first 40 days of hospitalization. The x-axis represents hospital days from admission (day 0) through day 40. The vertical dashed black line marks the date of transfer to our facility. Top panel: VRE culture results from urine (yellow) and stool (brown). Closed circles indicate positive cultures; open circles indicate negative cultures. Middle panel: *Enterococcus faecium* antimicrobial susceptibility results. Each column aligns with culture-positive days from the top panel. Top row indicates the testing platform. Subsequent rows represent individual antibiotics. Susceptibility is color-coded: green (susceptible), yellow (intermediate), red (resistant). MIC values are overlaid in white where available. The yellow asterisk on day 8 highlights an Etest result for linezolid with an MIC of 4 (intermediate). White spaces indicate unavailable results. Bottom panel: Timeline of resistant Gram-positive antibiotic administration. Each row corresponds to a specific antibiotic; black bars show days of administration. Fever days are shaded red; afebrile days are white. Fever information is unavailable prior to hospital transfer.

Three days after completing consolidation chemotherapy, she collapsed at home and presented in shock. She was found to have *Escherichia coli* bacteremia and a concurrent norovirus gastroenteritis requiring vasopressor support, mechanical ventilation, and treatment for disseminated intravascular coagulation. Her hospitalization was further complicated by neutropenic fevers in the setting of *Candida parapsilosis* fungemia and methicillin-resistant *Staphylococcus aureus* bacteremia. A urine culture obtained by straight catheterization on hospital day (HD) 8 grew *E. faecium* (10,000–25,000 CFU/mL) (Fig. 1). Susceptibility testing using the VITEK 2 platform with the Gram-positive susceptibility card (AST-GP67, CLSI M100 ED34 breakpoints) suggested vancomycin and linezolid resistance. Linezolid ETEST (bioMérieux) on the same isolate demonstrated a linezolid MIC of 4 µg/mL (intermediate by CLSI) (Table 1). Due to the discrepancy, VITEK 2 testing was repeated and again yielded linezolid resistance. MIC values from the VITEK 2 testing on this sample were not available.

**TABLE 1:** Vancomycin-resistant *Enterococcus faecium* microbiology results^*a*^

Hospital day	8	9	10	31
Source	Urine	Rectal swab	Urine	Urine
CFU/mL	10,000-25,000		4,000	50,000
Machine	VITEK 2	BD Phoenix	BD Phoenix	BD Phoenix

Antibiotic	Interpretation	MIC (μg/mL) | Interpretation	MIC (μg/mL) | Interpretation	MIC (μg/mL) | Interpretation
Linezolid	R^*b*^	2 | S	2 | S	≥8 | R
Vancomycin	R	>32 | R	>32 | R	>32 | R
Chloramphenicol		8 | S	8 | S	16 | I
Daptomycin		1 | S	4 | S	2 | S
Ciprofloxacin	R			
Levofloxacin	R		>4 | R	>4 | R
Penicillin	R	>16 | R	>16 | R	>16 | R
Tetracycline	R		>8 | R	>8 | R
Gentamicin–synergy			≤500 | S	≤500 | S
Minocycline		>8 | R	>8 | R	8 | I
Doxycycline			8 | I	4 | S
Ampicillin		>8 | R	>8 | R	>8 | R
Streptomycin–synergy			>1000 | R	>1000 | R
Quinupristin/dalfopristin	I			
Nitrofurantoin	R			

^*a*^ This table gives *Enterococcus faecium* microbiology results, including day of collection, source, CFU/mL isolated, testing machine, and susceptibility results. MIC values were not available from the transferring hospital microbiology laboratory where the urine isolate from HD 8 was collected.

^*b*^ Etest was also conducted for linezolid for HD8 urine isolate with MIC value of 4 which is intermediate by CLSI M100 ED34 breakpoints.

She was transferred to a tertiary pediatric hospital on HD 9 for ongoing multiorgan dysfunction management. At transfer, she was receiving linezolid (600 mg IV every 12 h), meropenem (renally dosed; 2 g IV every 12 h), and voriconazole (200 mg IV every 12 h).

On HD 10, a repeat urine culture from the indwelling catheter grew *E. faecium* (4,000 CFU/mL) (Fig. 1). Phoenix M50 Automated Microbiology System susceptibility testing (EpiCenter Version V752B/V7.31A) showed vancomycin resistance but linezolid susceptibility (MIC 2 µg/mL). Linezolid therapy was continued for presumed VRE pyelonephritis.

During a fever evaluation on HD31, an indwelling catheter urine culture grew *E. faecium* (50,000 CFU/mL) (Fig. 1). Phoenix testing now showed vancomycin resistance with concurrent linezolid nonsusceptibility (MIC ≥8 µg/mL) and intermediate chloramphenicol susceptibility (MIC 16 µg/mL). Linezolid was discontinued, and daptomycin (10 mg/kg/dose IV every 24 h) was initiated for 7 days.Subsequent urine cultures were negative for *E. faecium*.

She remained neutropenic with absolute neutrophil count <500 cells/µL until HD45 despite granulocyte colony-stimulating factor and 11 granulocyte transfusions. Her course was complicated by multiple other infections requiring broad-spectrum antimicrobial therapy. She ultimately received over 8 weeks of cumulative daptomycin therapy initially for her *E. faecium* urinary tract infection and subsequently for empiric Gram-positive coverage. She was discharged to a rehabilitation facility after approximately 6 months of hospitalization.

## DISCUSSION

This case illustrates the importance of linezolid resistance during treatment of a vancomycin-resistant *E. faecium* urinary tract infection in an immunocompromised host. Extensive antimicrobial exposure due to persistent infection in the setting of profound neutropenia and clinical illness may have selected for resistant organisms.

*E. faecium* resistance interpretation was complicated by discrepancies between testing platforms and the inherent susceptibility testing error of one dilution standard (21). All results were interpreted using CLSI M100 ED34 breakpoints, where linezolid MIC >2 µg/mL is nonsusceptible (4 µg/mL = intermediate; ≥8 µg/mL = resistant). By comparison, EUCAST v14 guidelines define susceptible as ≤4 µg/mL and resistant as >4 µg/mL, without an intermediate category; these definitions remain unchanged in CLSI ED35 and EUCAST v15. Pre-transfer VITEK 2 results suggested linezolid resistance, but manual ETEST categorized the isolate as intermediate. Per the transferring hospital's microbiology laboratory, it is not standard practice to perform repeat testing. Post-transfer Phoenix testing initially indicated susceptibility, followed by resistance later in hospitalization.

Automated susceptibility testing such as VITEK 2 and Phoenix provides rapid results but has limited accuracy for linezolid susceptibility testing in *Enterococci* (24). In one study of 100 *E. faecium*/*E. faecalis* isolates (including 38 with PCR-confirmed resistance genes), ETEST achieved the highest categorical agreement (87%) with broth microdilution using EUCAST breakpoints on day 1, compared to VITEK 2 (79%) and Phoenix (64%) (24). Extending incubation to >42 h yielded greater accuracy and precision compared with 18 h, with ETEST reaching 92% categorical agreement on day 2 using EUCAST breakpoints. Longer incubation is not an option for Phoenix or VITEK 2. None of the three methods reached the recommended ≥90% agreement using CLSI breakpoints. Every method yielded high rates of false-susceptible results, but this was improved using CLSI breakpoints versus EUCAST breakpoints. While manual methods like ETEST can be used for additional confirmation, they are not universally available and often not employed unless discrepancies are suspected (19, 25). Diagnostic uncertainty or misclassification may delay appropriate therapy, prolong ineffective treatment, or inappropriately limit therapeutic options.

Linezolid resistance in *E. faecium* commonly arises from mutations in domain V of the 23S rRNA gene, particularly the G2576T mutation, which reduces drug binding affinity (14). Some 23S rRNA mutations, including G2576T, confer resistance to both linezolid and chloramphenicol, and the presence of two mutations can have synergistic effects (26). Resistance can also arise from transferable genes such as *optrA* and *poxtA* (ribosomal protection) and *cfr* (23S rRNA methylation), which mediate cross-resistance to both drugs (14, 27). Both linezolid and chloramphenicol target the 50S ribosomal subunit, albeit at distinct sites (7, 9). Acquisition of these resistance mechanisms limits therapeutic options, necessitating reliance on agents like daptomycin, tigecycline, or quinupristin-dalfopristin—each with their own limitations, particularly with emerging daptomycin resistance in *E. faecium* (28). One report demonstrated co-localization of several of these resistance genes (*optrA* and *cfr* (D) in addition to *vanA*) on a single linear plasmid in *E. faecium* (29). In our patient, the progressive increase in chloramphenicol MIC alongside linezolid resistance raises concern for ribosomal target modification or protection, although molecular confirmation was not performed.

The identification of linezolid resistance in this patient’s VRE isolate necessitated a switch to intravenous daptomycin as the remaining active antimicrobial. In this case, the patient’s critical illness and clinical complexity required ongoing inpatient care regardless of administration route. However, for other patients, loss of linezolid can remove one of the few orally bioavailable options for resistant Gram-positive infections, limiting the feasibility of step-down therapy and discharge. More broadly, a patient’s history of multi-drug resistant bacteria can lead to “resistance entrapment,” where increasing antimicrobial resistance progressively restricts available therapeutic options. This case underscores both the clinical impact of losing linezolid as an option and the broader need for more agents active against *Enterococcus*.

This case demonstrates the complex interplay between antimicrobial resistance, diagnostic uncertainty, and clinical decision-making in the management of multidrug-resistant *E. faecium* infections in immunocompromised hosts. The prolonged hospitalization, persistent neutropenia, and frequent antimicrobial exposures created an ideal environment for resistance selection, while platform-to-platform variability in susceptibility testing complicated interpretation and clinical decision-making. These challenges highlight the importance of confirmatory testing when results are borderline or unexpected, careful integration of microbiologic data into empiric therapy decisions, and close collaboration between clinical teams and microbiology laboratories. Though molecular characterization was not available in this case, improved rapid resistance gene detection could improve reliability of resistance prediction to guide therapeutic decisions. This case underscores the need for improved diagnostic accuracy for *Enterococcus* resistance alongside urgent development of new antimicrobials to expand *Enterococcus* treatment options.

## Data Availability

All data relevant to this case report are included within the article. Additional anonymized information is available from the corresponding author upon reasonable request.
